# Alterations in intestinal microbiota and metabolites in individuals with Down syndrome and their correlation with inflammation and behavior disorders in mice

**DOI:** 10.3389/fmicb.2023.1016872

**Published:** 2023-02-23

**Authors:** Shaoli Cai, Jinxin Lin, Zhaolong Li, Songnian Liu, Zhihua Feng, Yangfan Zhang, Yanding Zhang, Jianzhong Huang, Qi Chen

**Affiliations:** ^1^Biomedical Research Center of South China, Fujian Normal University, Fuzhou, Fujian, China; ^2^Fujian Key Laboratory of Innate Immune Biology, Fujian Normal University, Fuzhou, Fujian, China; ^3^College of Life Sciences, Fujian Normal University, Fuzhou, Fujian, China; ^4^Institute of Animal Husbandry and Veterinary Medicine, Fujian Academy of Agricultural Sciences, Fuzhou, Fujian, China

**Keywords:** Down syndrome, cytokines, intestinal microbiota, metabolomics, inflammation

## Abstract

The intestinal microbiota and fecal metabolome have been shown to play a vital role in human health, and can be affected by genetic and environmental factors. We found that individuals with Down syndrome (DS) had abnormal serum cytokine levels indicative of a pro-inflammatory environment. We investigated whether these individuals also had alterations in the intestinal microbiome. High-throughput sequencing of bacterial 16S rRNA gene in fecal samples from 17 individuals with DS and 23 non-DS volunteers revealed a significantly higher abundance of *Prevotella*, *Escherichia*/*Shigella*, *Catenibacterium*, and *Allisonella* in individuals with DS, which was positively associated with the levels of pro-inflammatory cytokines. GC-TOF-MS-based fecal metabolomics identified 35 biomarkers (21 up-regulated metabolites and 14 down-regulated metabolites) that were altered in the microbiome of individuals with DS. Metabolic pathway enrichment analyses of these biomarkers showed a characteristic pattern in DS that included changes in valine, leucine, and isoleucine biosynthesis and degradation; synthesis and degradation of ketone bodies; glyoxylate and dicarboxylate metabolism; tyrosine metabolism; lysine degradation; and the citrate cycle. Treatment of mice with fecal bacteria from individuals with DS or *Prevotella copri* significantly altered behaviors often seen in individuals with DS, such as depression-associated behavior and impairment of motor function. These studies suggest that changes in intestinal microbiota and the fecal metabolome are correlated with chronic inflammation and behavior disorders associated with DS.

## Introduction

Down syndrome (DS), a genetic disease caused by trisomy of chromosome 21, was first described by Doctor John Langdon Down in 1866, and is characterized by abnormal brain development, including the reduction of total brain volume, specifically in the cortical, hippocampal, and cerebellar areas (Zigman, 2013). DS occurs in approximately 1–2 per 1,000 live births across the world (Parker et al., 2010; Marlow et al., 2021; Osborne et al., 2021), with a prevalence of 1/800 worldwide, 1/500 in the United States, and 1.4/1,000 in China (Xiao et al., 2022). Approximately 50% of babies with DS die *in utero*; the survival rate of infants with DS is approximately 90% (Best et al., 2018). Due to numerous impairments, individuals with DS may suffer a low quality of life and require costly, life-long care; it has been estimated that DS is associated with a financial burden of US$60,000 (Bull, 2020). Since chromosome 21 encodes many genes, the course of DS is polygenic and complex. Individuals with DS are at high risk of congenital heart disease, developmental delay, leukemia, obesity, obstructive sleep apnea, and abnormal myelopoiesis and inflammatory responses (Khalid-Raja and Tzifa, 2016). Many studies have reported immune system impairments in DS, with high levels of pro-inflammatory and low levels of anti-inflammatory cytokine production (Huggard et al., 2020). Suppression of pro-inflammatory cytokines in individuals with DS may reduce the risk of comorbid conditions and improve quality of life.

Intestinal microbiota are a colonizing flora of microorganisms in the human body, comprising anaerobic bacteria, protozoa, fungi, and archaeas. Among these, bacteria are the most prominent microorganism, with 150-fold more genes than the human genome (Qin et al., 2010). Bacteria within the microbiome secrete a range of enzymes that modulate diverse host functions, such as metabolism of indigestible carbohydrates, production of vitamins, reproduction and differentiation of the intestinal epithelium, regulation of the immune system, and maintenance of intestinal homeostasis (Zimmermann et al., 2019; Casertano et al., 2021), which help hosts adapt various environments (Xiong, 2022). Clinical evidence has shown that the composition of intestinal microbiota is different between patients with various diseases and the healthy population (Bäumler and Sperandio, 2016). For example, gut microbial alterations are associated with cognitive impairment and Aβ load in older adults (Wu et al., 2022), and the composition of the gut microbiota of patents with Alzheimer’s disease (AD) differs from that of healthy controls at the taxonomic level. Perturbation of intestinal microbiota composition has also been linked to accelerated development of inflammation in people suffering from memory impairment (Wang et al., 2021).

Metabonomics is the study of changes in metabolism in response to pathophysiological states or exogenous substances. For example, the composition of serum saturated fatty acids and unsaturated fatty acids was shown to vary between individuals with AD and healthy volunteers (Wang et al., 2012), and analysis of the urinary metabolome revealed differences in metabolites between patients with interstitial cystitis and healthy people (Kind et al., 2016). Interestingly, many studies have suggested that changes in intestinal microbiota composition are associated with alterations in host metabolites in the gut (Guo et al., 2020).

The current study explored the production of pro-inflammatory cytokines, intestinal microbiota, and fecal metabolites in individuals with DS and non-DS volunteers using multiparameter flow cytometry analysis, high-throughput sequencing of the intestinal microbiome, and gas chromatography time-of-flight mass spectrometry (GC-TOF-MS)-based fecal metabolomics. The analysis revealed differences in the intestinal microbiota of individuals with DS and non-DS volunteers, a correlation between gut microbial flora/fecal metabolite markers and pro-inflammatory cytokines in DS individuals, and association of the DS microbiome with DS-related behaviors in mice. These studies may provide insights into DS pathophysiology and inform the development of new therapeutics for individuals with DS.

## Materials and methods

### Study participants

A total of 40 participants were recruited: 23 non-DS volunteers (HC) and 17 individuals with DS (Supplementary Table 1). Participants were recruited from the Fuzhou Second Social Welfare Home (Fuzhou, China). All the DS individuals had a congenital cognitive delay, while all non-DS volunteers had a physical disability. Ethical approval for this study was provided by the NHS Health Research Authority (REC reference: 15/SW/0354) and informed consent was obtained from all volunteers or their legal guardians prior to enrollment in the study.

### Sample collection

All samples were collected from participants on the same day (03. 23. 2017). Whole blood was collected by venipuncture after a 12 h fast and stored at 25°C for 0.5 h. Samples were centrifuged at 3,000 *g* for 20 min to collect the serum, which was stored at −80°C until use. Fecal samples were collected in sterile urine containers and stored at −80°C until use.

### Measurement of cytokine biomarkers in serum

Serum interleukin-9 (IL-9), interleukin-1β (IL-1β), macrophage inflammatory protein-1α (MIP-1α), angiogenin, granulocyte colony-stimulating factor (G-CSF), interleukin-1α (IL-1α), monocyte chemoattractant protein 1 (MCP-1), macrophage inflammatory protein-1β (MIP-1β), immunoglobulin E (IgE), interleukin-6 (IL-6), fractalkine, interleukin-8 (IL-8), tumor necrosis factor-α (TNF-α), monokine induced by interferon-gamma (MIG), rantes, and granzyme B were analyzed by multiparameter flow cytometry using the corresponding antibodies from BD Biosciences (East Rutherford, NJ, United States) and the BD FACSymphony^™^A5 (BD Biosciences).

### Intestinal microbiota analysis

Genomic DNA was isolated from frozen fecal samples using a DNA isolation kit (MoBio, Carlsbad, CA, United States) and quantified using a NanoDrop ND-1000 spectrophotometer (Thermo Fisher Scientific, Waltham, MA, United States). 16S rRNA genes (V3-V4 region) were PCR amplified using the forward primer 341F (5′-ACTCCTACGGGRSGCAGCAG-3′) and reverse primer 806R (5′-GGACTACVVGGGTATCTAATC-3′), purified using Agencourt AMPure magnetic purification beads (Beckman Coulter, Brea, CA, United States), and run on 2% agarose gel. High-throughput sequencing of the PCR products was carried out on the PacBio RS II platform and analyzed at Ruiyi Biotechnology Co., Ltd. (Hangzhou, China).

Raw data from high-throughput sequencing were demultiplexed and quality filtered using the QIIME2 platform, then used to assemble operational taxonomic units (OTUs) to define species, genus, or class of bacterial communities by UCLUST algorithm (an exceptionally fast sequence clustering program for nucleotide and protein sequences) with a threshold of 97%. Principal component analysis (PCA) plots were generated using R software (v 4.1.2). STAMP (Ver. 2.1.3) software was applied to identify intestinal microbial phylotypes.

### Fecal sample preparation for metabolomic analysis

Fecal samples (100 mg) were mixed with 500 μL of extract solution (methanol:chloroform = 3:1) and 20 μL of internal standard (L-2-chlorophenylalanine), followed by ultrasonic treatment on ice. The mixture was centrifuged at 13,000 rpm for 20 min at 4°C. The supernatant (400 μL) was transferred to an Eppendorf tube, dried in a vacuum concentrator at 30°C, and dissolved in 80 μL of methoxyamine hydrochloride (in pyridine, 20 mg/mL). Samples were placed at 75°C for 30 min, then 100 μL of pyridine and N,O,-bis-(trimethylsilyl) trifluoroacetamide (BSTFA) (containing 1% trimethylchlorosilane, v/v) was added and the samples were incubated at 70°C for 90 min. After cooling to 25°C, the samples were mixed well with 8 μL of fatty acid methyl esters (FAME)s and used for GC-TOF-MS analysis.

### Metabolomic analysis with GC-TOF-MS

GC-TOF-MS analysis was carried out using a gas chromatograph system coupled with a Pegasus HT time-of-flight mass spectrometer. The system utilizes a DB-5 MS capillary column coated with 5% diphenyl cross-linked with 95% dimethylpolysiloxane (30 m × 250 μm × 0.25 μm, J&W Scientific, United States). An analyte (1 μL) was injected in splitless mode. Helium was used as the carrier gas, the front inlet purge flow was 3 mL/min, and the gas flow rate through the column was 20 mL/min. The initial temperature was kept at 50°C for 1 min, then raised to 310°C at a rate of 10°C/min and kept for 9 min at 310°C. The injection, transfer line, and ion source temperatures were 280, 270, and 220°C, respectively. The energy was −70 eV in electron impact mode. The mass spectrometry data were acquired in full-scan mode with the m/z range of 50–500 at a rate of 20 spectra/s after a solvent delay of 312 s.

The raw data from GC-TOF-MS were processed using Progenesis software (v 3.0) for peak detection, filtering, denoising, alignment, and standardization. The resulting normalized data were evaluated by multivariate statistical analyses using R software (v 4.1.2), including PCA, partial least squares discriminant analysis (PLS-DA), and orthogonal partial least squares discriminant analysis (OPLS-DA). Potential biomarkers were selected based on the VIP (variable importance plots) value and *p* values were determined by two-tailed *t*-test. A VIp value more than 1.0 and *P* value less than 0.05 were considered statistically significant. Heatmaps were constructed based on the area of potential biomarkers using a random forest algorithm with TBtools software. Pathway enrichment analysis of the potential biomarkers was performed using the website MetaboAnalyst^1^ based on the KEGG database.^2^ Correlation between the potential biomarkers and pro- and anti-inflammatory cytokines was determined using R software (v4.1.2) and Cytoscape (v3.9.0).

### Animals and behavioral studies

Twenty-four germ-free (GF) C57BL/6 mice (6 weeks of age) were supplied by the Chinese Academy of Medical Sciences (Beijing, China) and used for behavioral testing following treatment with fecal bacteria derived from individuals with DS and non-DS volunteers, as well as a preparation of *Prevotella copri.* All animal experimental procedures adhered to guidelines approved by the Chinese Academy of Medical Sciences [Permit No. SYXK (Beijing)-2018–0019].

Mice were housed in a standard room at 22 ± 1°C with a humidity of 55 ± 5%, under a 12 h light/dark cycle at the Chinese Academy of Medical Sciences Animal Facility. After acclimation for 7 days, the mice were randomly divided into four groups of six mice and received the following treatments twice a week for 42 days. (1) The control group was treated with intragastric gavage of 100 μL phosphate-buffered saline. (2) The HC group was treated with intragastric gavage of 100 μL of fecal bacteria pooled from 23 non-DS volunteers. (3) The DS group was treated with intragastric gavage of 100 μL of fecal bacteria pooled from 17 participants with DS. (4) The *Prevotella* group was treated with intragastric gavage of 100 μL of *Prevotella copri* (10^8^ CFU). After 42 days of treatment, the mice were underwent a series of behavior tests including sucrose preference test, open field test, and forced swimming test, as described below. All mice were then anesthetized with isoflurane and blood samples were collected by heart puncture after a 12-h fast following the accomplishment of all behavior tests.

### Sucrose preference test

Depression is often associated with DS (Thom et al., 2021). The sucrose preference test was used to measure anhedonia-like symptoms (indicating depression-like behavior) over a 48-h period. Animals were acclimated to two identical water bottles on their cages for 3 days. Then, each mouse was given two bottles (A: water and B: 2% sucrose solution). To avoid a locational bias, we changed the position of bottles A and B every 12 h. The amount of the sucrose solution or water consumed was measured by weighing the bottles before and after the test. The percentage of sucrose preference was calculated as an index of depression-like behavior.

### Open field test

Basic motor skills, movement disorders, and abnormal gait and posture are correlated with cognitive limitations and abnormal sensorimotor integration found in DS (Carvalho and Vasconcelos, 2011). Therefore, the open field test was performed to assess locomotor activity and exploratory behavior of mice. Briefly, mice were placed individually in 40 cm × 60 cm × 50 cm box with the floor divided into 25 smaller rectangular units. The total time spent in the central zone and total distance crossed by each mouse were recorded every 5 min for 30 consecutive days. Between each test, the apparatus was cleaned with 75% ethanol solution to eliminate possible odors left by other mice.

### Forced swimming test

The forced swimming test is a non-invasive behavioral test often used for evaluation of depression in rodents (Can et al., 2012). Therefore, forced swimming tests were carried out using a previously reported protocol (Hirano et al., 2009). Each mouse was placed into a transparent cylinder (20 cm × 20 cm × 40 cm) filled with water (depth of water: 30 cm and temperature: 25 ± 1°C). Immobility time was recorded during the last 4 min of the 6-min testing period.

### Serum biochemical analysis

Blood samples collected from the mice were placed at room temperature for 2 h then centrifuged (6,000 rpm, 25°C, 15 min). Serum C-reactive protein (CRP), lipopolysaccharide (LPS), and corticosterone levels were measured using commercially available kits according to the manufacturer’s instructions (Baiaolaibo Technology Co., Beijing, China).

### Statistical analysis

Experimental results are expressed as the mean ± SEM using Prism 7.0 (GraphPad Software, San Diego, CA, United States). Statistical analysis was carried out using one-way analysis of variance (ANOVA), followed by Tukey test using SPSS 20.0 (IBM, Chicago, IL, United States) unless otherwise indicated. Statistically significant differences are indicated as *p < 0.05, and **p < 0.01.

In the meantime, we used LDA Effect Size (LEfSe) analysis and rank sum test to analyze the differences between groups and to find out the species that had significant differences between groups. For the analysis results of the rank sum test, we performed False Discovery Rate (FDR) correction on the value of *p*, and selected the different species according to the corrected value of *p*.

## Results

### Cytokine levels are shifted in individuals with DS

**Figure 1:**
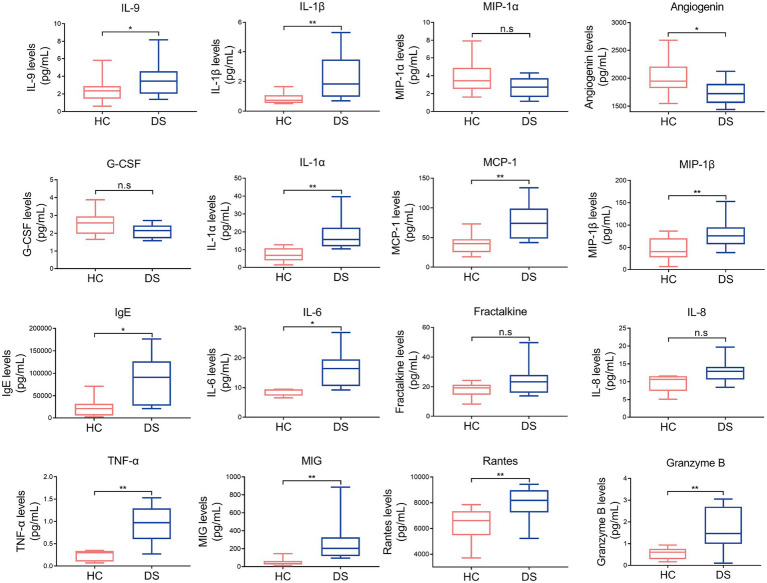
Pro-inflammatory and anti-inflammatory cytokines in individuals with DS and non-DS volunteers (HC). Serum levels of IL-9, IL-1β, MIP-1α, angiogenin, G-CSF, IL-1α, MCP-1, MIP-1β, IgE, IL-6, fractalkine, IL-8, TNF-α, MIG, rantes, and granzyme B were analyzed by multiparameter flow cytometry. *p < 0.05 and **p < 0.01 when comparing DS with the non-DS group.

Chronic inflammatory conditions and autoimmunity are common features of DS, with previous studies reporting high levels of circulating pro-inflammatory cytokines and low levels of anti-inflammatory cytokines. Therefore, we examined the serum levels of IL-9, IL-1β, IL-1α, MCP-1, MIP-1β, IgE, IL-6, TNF-α, MIG, rantes, granzyme B, angiogenin MIP-1α, G-CSF, fractalkine, and IL-8 in individuals with DS and non-DS volunteers. As shown in Figure 1, the levels of serum IL-9, IL-1β, IL-1α, MCP-1, MIP-1β, IgE, IL-6, TNF-α, MIG, rantes, and granzyme B were all significantly increased in the DS group compared with the non-DS group (*p* < 0.05). The level of serum angiogenin was significantly reduced in the DS group compared with the non-DS group (*p* < 0.05). No significant differences were seen in the serum levels of MIP-1α, G-CSF, fractalkine, and IL-8 between the DS group and the non-DS group (*p* > 0.05). These results indicate the presence of a distinct pro-inflammatory cytokine profile in individuals with DS.

### Composition and diversity of intestinal microbiota are altered in individuals with DS

**Figure 2:**
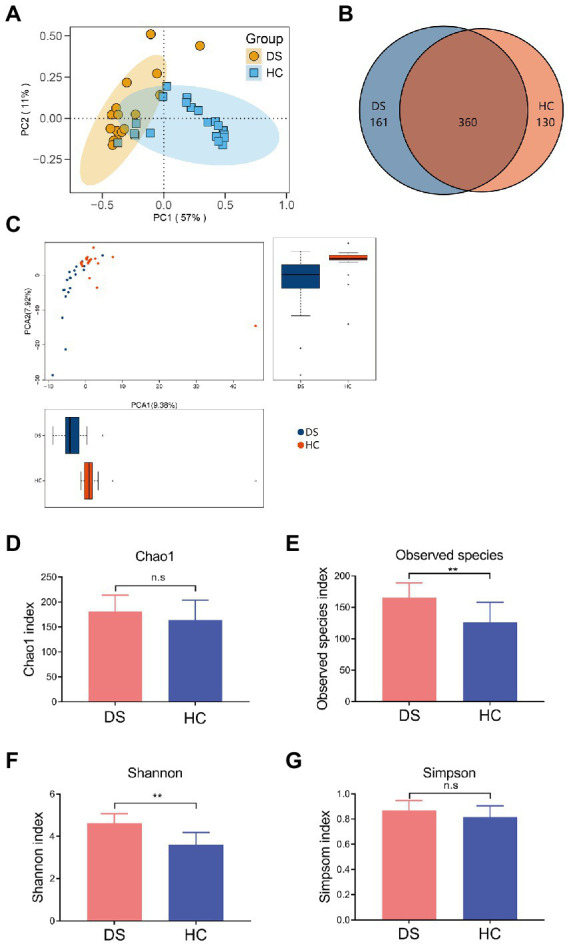
Differences in the intestinal microbiota composition and diversity in the non-DS and DS groups. 16S rRNA sequencing data was used to assemble operational taxonomic units (OTUs). **(A)** PCA plot of intestinal flora. **(B)** Venn diagram of intestinal microbiota. **(C)** Analyses of intestinal flora based on OTUs. **(D–G)** Alpha diversity was used to assess the flora diversity and distribution, including the Chao1 index **(D)**, observed species index **(E)**, Shannon index **(F)**, and Simpson index **(G)**. **A significant difference (*p* < 0.01) in the observes species of intestinal microbiota was seen in individuals with DS and non-DS volunteers. HC, non-DS volunteers (or non-DS group); DS, individuals with DS (or DS group).

The intestinal microbiota play a vital role in the inflammatory response. Thus, we examined the composition of intestinal microbiota in the non-DS and DS groups using high-throughput sequencing analysis. A PCA biplot of the intestinal microbiota structure revealed an apparent separation between the non-DS and DS groups (Figure 2A). The first principal component (PC1) and the second principal component (PC2) accounted for 57 and 11% of the total variation, respectively, i.e., the intestinal microbiota in the non-DS group was mainly distributed in the second and third quadrants, whereas the intestinal microbiota in the DS group was mainly distributed in the first and fourth quadrants. As shown in Figure 2B, there were a total of 651 OTUs in the non-DS and DS groups, with each OTU representing a single species. Among these, 360 OTUs overlapped in the non-DS and DS groups. The DS group exhibited 161 unique OTUs, whereas the non-DS group displayed 130 unique OTUs. Thus, individuals with DS had significantly higher fecal microbial OTUs compared with non-DS volunteers. As seen in Figure 2C, the distribution of fecal flora was relatively concentrated in the non-DS group, and relatively scattered in the DS group, indicating that the individual difference ratio in fecal flora in non-DS volunteers was small, with greater variation among individuals with DS. The fecal flora of the DS group tended to move away from that of the non-DS group, indicating variations in microbiome abnormality characteristics that may be linked to variations in DS severity. In summary, the intestinal flora of individuals with DS was significantly different from that of non-DS volunteers, with clear differences in intestinal microbiota diversity.

Next, we conducted alpha diversity analyses to further assess the species diversity and distribution in the two study groups, calculating the Chao1 index, observed species index, Shannon index, Simpson index, and PD whole Tree index. The alpha diversity index of each sample was calculated using QIIME software, and a corresponding dilution curve was generated. The Chao algorithm was used to estimate the OTU index (the total number of species) in the flora. As shown in Supplementary Table 2, compared with the non-DS group, the Chao value of intestinal bacteria in individuals with DS was higher, indicating that the overall number of intestinal microorganisms in individuals with DS was more abundant. The intestinal flora diversity of individuals with DS (Simpson = 0.8447) was slightly higher than that of the non-DS group (Simpson = 0.8067), but the difference was not significant (Supplementary Table 2). The observed species and Shannon index in the DS group were significantly higher than that in the non-DS group (*p* < 0.01; Figures 2E,F); however, there was no significant difference in Chao1 and the Simpson index between the non-DS and DS groups (*p* > 0.05; Figures 2D,G).

**Figure 3:**
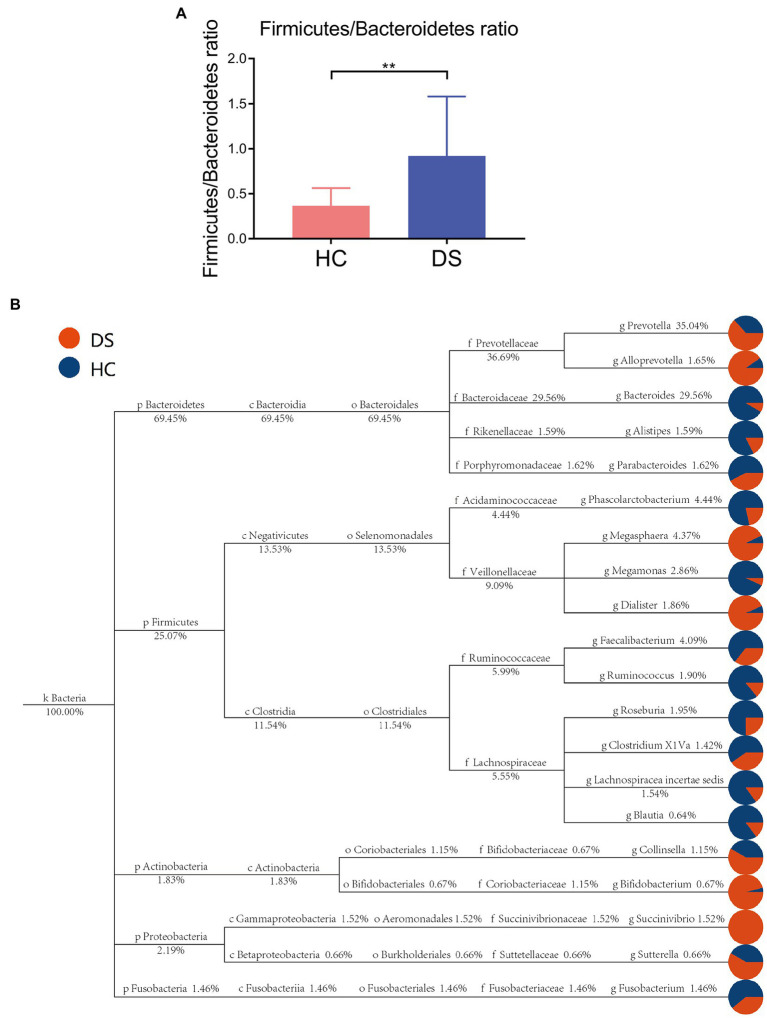
Taxon-based analysis of the microbiome in the DS and non-DS groups. **(A)** A significant difference was observed (**p < 0.01) in the ratio of *Firmicutes* and *Bacteroidetes* in the intestinal microbiota of the non-DS and DS groups. **(B)** The classification and abundance of intestinal microbiota from individuals with DS and non-DS volunteers. Different colors represent classification levels, and the size of the circle represents the relative abundance of the classification. The number below the taxonomic name indicates the relative percentage abundance. HC, non-DS volunteers (or non-DS group); DS, individuals with DS (or DS group).

Taxon-based analysis at the phylum level revealed differences in specific bacterial phylotypes between the non-DS and DS groups (Figures 3A,B). *Firmicutes* and *Bacteroidetes* are the primary phyla in gut, and their ratio is often associated with host health status. We found that the ratio of *Firmicutes* to *Bacteroidetes* was significantly elevated in the DS group compared with the non-DS group (*p* < 0.01). At the family level, *Prevotellaceae* was most abundant in the feces of individuals with DS, followed by *Ruminococcaceae*, *Veillonellaceae*, *Lachnospiraceae*, and *Bacteroidaceae,* suggesting that *Prevotellaceae* may be a characteristic intestinal bacteria of individuals with DS at the family level (Supplementary Figure 1). In contrast, *Bacteroidaceae* was the most abundant in the feces of non-DS volunteers, followed by *Prevotellaceae, Lachnospiraceae, Ruminococcaceae*, and *Acidaminococcaceae*. Therefore, *Bacteroidaceae* may be the characteristic intestinal bacteria in the non-DS group at the family level (Supplementary Figure 1).

**Figure 4:**
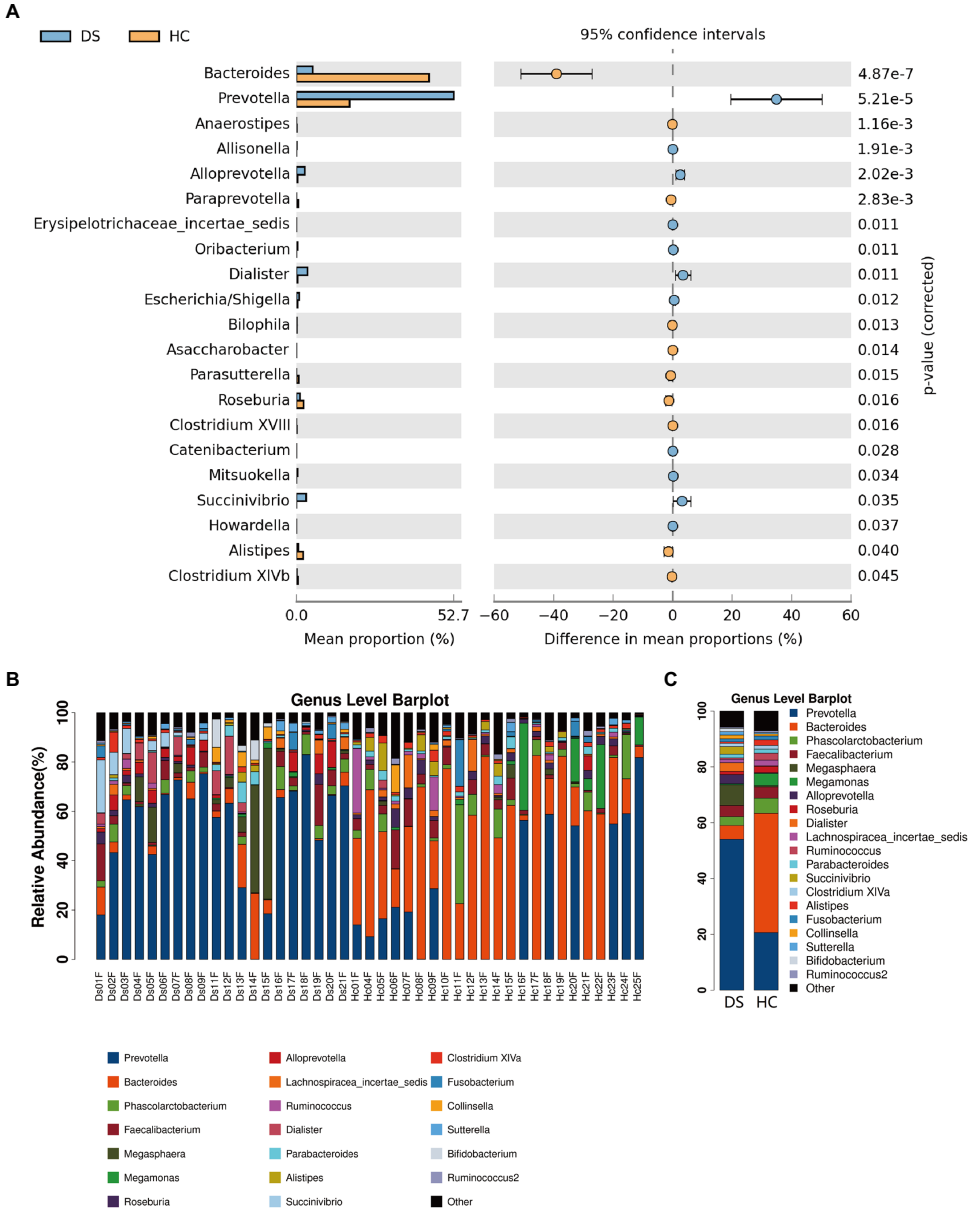
Differences in microbial composition between individuals with DS (DS) and non-DS volunteers (HC) at the genus level. **(A)** Mean proportion (left) and difference in mean proportion (right) of each genus present in the microbiome of DS and non-DS groups. **(B)** Fecal microbial richness of each DS and non-DS volunteer at the genus level. **(C)** Mean composition and richness of fecal microbiota in individuals with DS and non-DS volunteers.

At the genus level, the fecal microbiome of individuals with DS was enriched in *Prevotella, Bacteroides, Faecalibacterium, Alloprevotella, Megasphaera*, and *Dialister* (Figure 4A). By comparison, the fecal microbiome of the non-DS group was enriched for *Bacteroides, Prevotella, Megasphaera, Phascolarctobacterium, Faecalibacterium, Roseburia*, and *Lachnospiracea incertae sedis*. The relative abundance of 20 genera was significantly different between individuals with DS and non-DS volunteers (Figures 4B,C). The relative abundance of *Bacteroides, Anaerostipes, Paraprevotella, Bilophila, Asaccharobacter, Parasutterella, Roseburia, Clostridium XVIII, Alistipes*, and *Clostridium XIVb* was significantly lower in the DS group compared with the non-DS group. The relative abundance of *Prevotella, Allisonella, Alloprevotella, Erysipelotrichaceae incertae sedis, Oribacterium, Dialister, Escherichia/Shigella, Catenibacterium, Mitsuokella, Succinivibrio*, and *Howardella* was significantly higher in the DS group. Therefore, *Prevotella* and *Bacteroides* emerged as the characteristic bacteria of individuals with DS and non-DS volunteers, respectively. These data offer potential microbiome-based biomarkers of DS that may be linked to gut and overall health.

According to the flora classification, among the top 20 genera, we set the abundance difference and evolutionary relationship of dominant species in single or multiple samples against the entire classification system (Figure 3B). This analysis confirmed *Prevotella* as the dominant bacterium in individuals with DS, with a relative abundance of 35.04%, while *Bacteroides* was the dominant bacteria in non-DS volunteers, with a relative abundance of 29.56%. Both *Prevotella* and *Bacteroides* belong to *Bacteroidetes* (plylum), *Bacteroidia* (class), and *Bacteroides* (order). *Bacteroides* can be further divided into *Prevotellaceae* (family)*, Prevotella* (genus) and *Bacteroidaceae* (family), *Bacteroides* (genus). This suggests that that the intestines of individuals with DS may provide a preferred environment for *Prevotella* rather than *Bacteroides*, and that the relative level of order *Bacteroides* bacteria may be a key marker of DS linked to severity.

We next used hierarchical clustering to reveal the arrangement of groups (clusters) of similar bacteria within the intestinal flora in the DS and non-DS groups. The top 20 genera were used for a comparative analysis. As seen in Supplementary Figure 2, at the genus level, the fecal flora of individuals with DS and non-DS people separated into two clusters with a few exceptions, suggesting distinct differences in the fecal microbiota between the two study groups. As shown in Supplementary Figure 3, a heatmap was generated to represent the relative abundance of each genus by color gradient and cluster the data according to the similarity of species or sample abundance. This analysis showed a similar result: *Prevotella* is the dominant bacterium in fecal samples of individuals with DS, whereas *Bacteroides* is the dominant bacteria in the non-DS group. Of note, in the heatmap of species distribution, some samples in one group were clustered outside another group. Nonetheless, there were significant differences in the expression of dominant bacteria between the two groups, with a clear shift in the composition of intestinal microbiota in individuals with DS.

In addition, we used linear discriminant analysis effect size (LEfSe) analysis to compare the two groups and identify any subgroups with significant differences in bacterial abundance at the order, family, and genus levels. As shown in Supplementary Figure 4, this analysis further confirmed differences between the two groups, with *Prevotella, Prevotellaceae,* and *Veillonellaceae* identified as the dominant bacterial flora in individuals with DS, and *Bacteroides, Bacteroidaceae,* and *Lachnospiracea* as the dominant bacterial flora in the non-DS group.

### Differential abundance of fecal metabolites in individuals with DS

The alteration of intestinal microbiota can lead to the changes in fecal metabolomics. The analytical tools used for detecting the fecal metabolites include GC-TOF-MS, nuclear magnetic resonance (NMR), and ultra-performance liquid chromatography-mass spectrometry (LC–MS) (Chen et al., 2020). Among these tools, we selected GC-TOF-MS due to its complete database, fast scanning rate, its high efficiency, and sensitivity (Ma et al., 2011). To explore the connection between intestinal microbiota and fecal metabolomics in individuals with DS, we performed fecal metabolomics-based GC-TOF-MS analyses followed by PCA, PLS-DA, and OPLS-DA analyses and compared fecal metabolic profiles between the non-DS and DS groups. PCA score scatter plot and volcano plot were used to illustrate differences in all metabolites in all samples (Supplementary Figure 5). A total of 35 substances were detected with highly significant differences between the two study groups (Supplementary Table 3). Compared with non-DS volunteers, 21 fecal metabolites were significantly higher in individuals with DS, indicated by the red section of the table (ID 1–21). A total of 14 fecal metabolites were significantly lower in individuals with DS compared to non-DS volunteers, indicated by the blue section of the table (ID 22–35).

**Figure 5:**
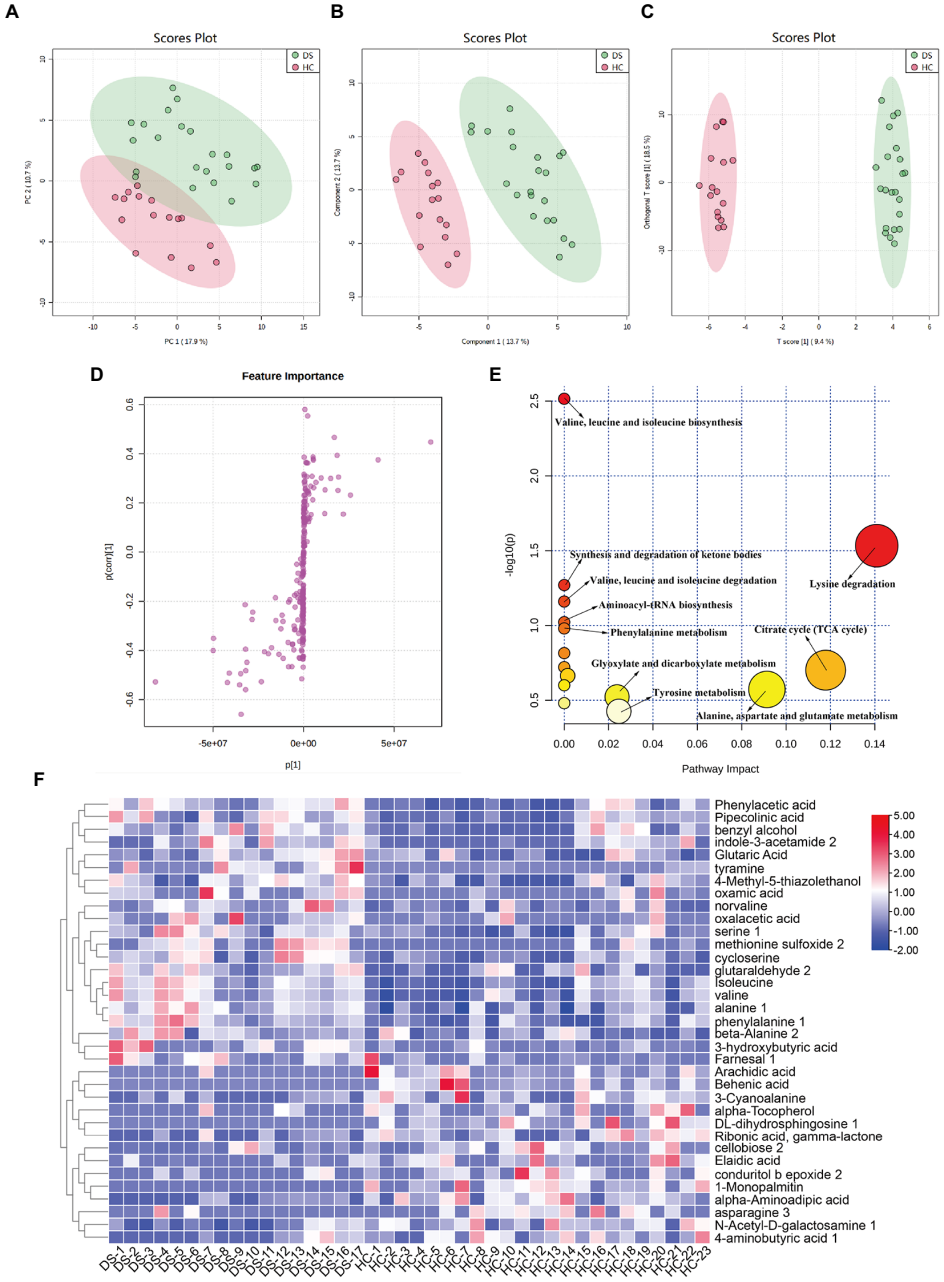
Differences in fecal metabolomic profiles and pathways in individuals with DS and non-DS volunteers. **(A)** Fecal metabolomic profiling by GC-TOF-MS. PCA score plot for the non-DS and DS groups. **(B)** PLS-DA score plot for the non-DS and DS groups. **(C)** OPLS-DA score plot for the non-DS and DS groups. **(D)** S-loading plot based on the OPLS-DA analysis of fecal metabolomics. **(E)** Heatmap of the relative abundance of significantly different metabolites (VIP > 1.0 and *p* < 0.05) between the non-DS (HC) and DS groups. **(F)** Metabolic pathway impact prediction based on the KEGG online database. The -ln(*p*) values from the pathway enrichment analysis are indicated on the horizontal axis, and the impact values are indicated on the vertical axis. HC, non-DS volunteer (or non-DS group); DS, individual with DS (or DS group).

As shown in Figure 5A, the metabolic spectra of two groups were separated by the PCA score plots, suggesting a significant difference in fecal metabolite profiles between the non-DS and DS groups. PLS-DA and OPLS-DA analyses of the fecal metabolome further illustrated significant separation between the non-DS group and DS group (Figures 5B,C). As illustrated in Figure 5D, the fecal metabolites in the load map located far from the center point were regarded as significantly changed (increased or decreased) in individuals with DS compared with non-DS volunteers, and were marked as potential biomarkers. As shown in Supplementary Table 3, a total of 35 potential biomarkers differed in feces between the non-DS and DS groups, including 21 metabolites that were increased and 14 that were decreased in the DS group compared with the non-DS group. As shown in Figure 5F, metabolites that were higher in the DS group than in the non-DS group included amino acids (serine, isoleucine, valine, alanine, phenylalanine, norvaline, cycloserine, and alanine); acids (phenylacetic acid, pipecolinic acid, glutaric acid, oxalacetic acid, 3-hydroxybutyric acid, and oxamic acid); amines (indole-3-acetamide, tyramine); alcohols (benzyl alcohol, and 4-methyl-5-thiazolethanol); and others (methionine sulfoxide, glutaraldehyde, and farnesal). Metabolites that were lower in the DS group than in the non-DS group included fatty acids (arachidic acid, behenic acid, alpha-tocopherol, elaidic acid, and 1-monopalmitin); amino acids (3-cyanoalanine, alpha-aminoadipic acid, and asparagine); carbohydrates (ribonic acid, cellobiose, N-acetyl-D-galactosamine, and 4-aminobutyric acid); and others (DL-dihydrosphingosine, gamma-lactone, and conduritol epoxide). Metabolic pathway enrichment analyses of these potential biomarkers (Figure 5E) suggested that valine, leucine, and isoleucine biosynthesis; synthesis and degradation of ketone bodies; valine, leucine, and isoleucine degradation; aminoacyl-tRNA biosynthesis; phenylalanine metabolism; glyoxylate and dicarboxylate metabolism; tyrosine metabolism; lysine degradation; citrate cycle (TCA cycle); and alanine, aspartate, and glutamate metabolism were different between the DS and non-DS groups.

### Cytokine levels are correlate with key microbial phylotypes and fecal metabolites

**Figure 6:**
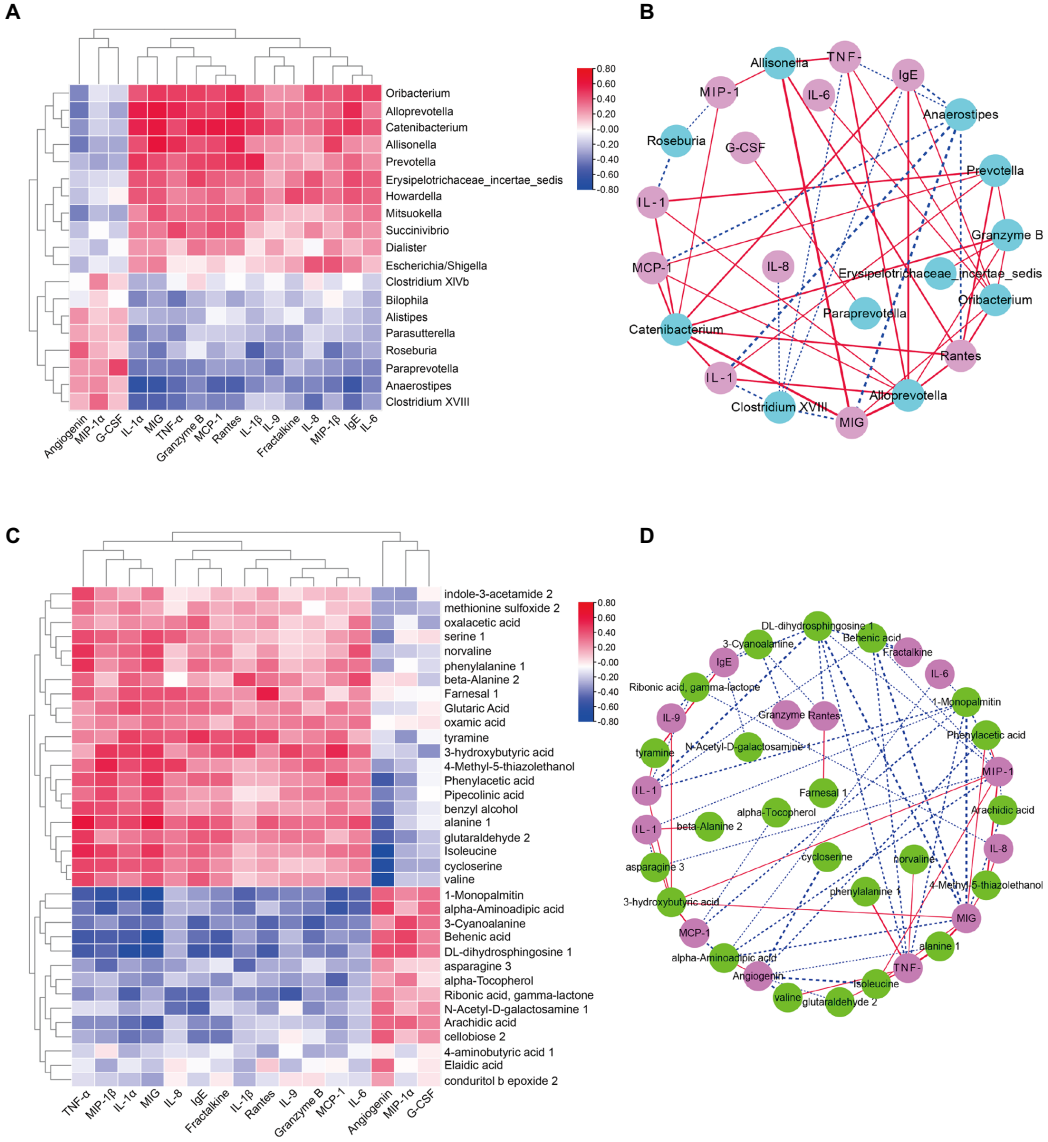
Correlation analyses of intestinal bacterial flora, metabolites, and cytokine levels in DS and non-DS groups. **(A)** Heatmap of Spearman’s correlation between the key intestinal bacterial phylotypes and cytokine levels. Red indicates a positive correlation and blue indicates a negative correlation. **(B)** Visualization of the correlation network based on partial correlation between the key intestinal bacterial phylotypes (teal) and cytokines (pink). Red lines indicate a positive correlation and blue lines indicate a negative correlation. The thicker the line, the stronger the correlation. **(C)** Heatmap of Spearman’s correlation between the significantly different metabolites and cytokine levels. Red indicates a positive correlation and blue indicates a negative correlation. **(D)** Visualization of the correlation network based on partial correlation between the significantly different metabolites (green) and cytokines (purple). Red lines indicate a positive correlation and blue lines indicate a negative correlation. The thicker the line, the stronger the correlation. HC, non-DS volunteers (or non-DS group); DS, individuals with DS (or DS group).

To further address whether the observed differences in intestinal microbiota and the fecal metabolome in the DS and non-DS groups are linked to differences in their cytokine profiles, we performed a heatmap and network study. The correlation between cytokines and key microbiota phylotypes is illustrated in Figures 6A,B. Genus *Oribacterium*, *Alloprevotella*, *Catenibacterium*, *Allisonella*, *Prevotella*, *Erysipelotrichaceae incertae sedis*, *Howardella*, *Mitsuokella*, *Succinivibrio*, *Dialister*, and *Escherichia*/*Shigella* were positively associated with the levels of serum IL-1α, MIG, TNF-α, granzyme B, MCP-1, rantes, IL-1β, IL-9, fractalkine, IL-8, MIP-1β, IgE, and IL-6, and negatively associated with the levels of serum angiogenin, MCP-1α, and G-CSF. However, genus *Bilophila*, *Alistipes*, *Parasutterella*, *Roseburia*, *Paraprevotella*, *Anaerostipes*, and *Clostridium* XVIII were negatively associated with the levels of serum IL-1α, MIG, TNF-α, granzyme B, MCP-1, rantes, IL-1β, IL-9, fractalkine, IL-8, MIP-1β, IgE, and IL-6, and positively associated with the levels of serum angiogenin, MCP-1α, and G-CSF. Intestinal microbiota-derived metabolites are reported to accelerate or prevent inflammation. Therefore, we used Spearman’s correlation analysis to further uncover the potential correlation between fecal metabolites and cytokines in individuals with DS (Figures 6C,D). Indole-3-acetamide 2, methionine sulfoxide 2, oxamic acid, serine 1, norvaline, phenylalanine1, beta-alanine 2, farnesal 1, glutaric acid, oxamic acid, tyramine, 3-hydroxybutyric acid, 4-methyl-5-thiazolethanol, phenylacetic acid, pipecolinic acid, benzyl alcohol, alanine 1, glutaraldehyde 2, isoleucine, cycloserine, and valine were positively associated with the levels of serum IL-1α, MIG, TNF-α, granzyme B, MCP-1, rantes, IL-1β, IL-9, fractalkine, IL-8, MIP-1β, IgE, and IL-6, and negatively associated with the levels of serum angiogenin, MCP-1α, and G-CSF. In contrast, 1-monopalmitin, alpha-aminoadipic acid, 3-cyanoalanine, behenic acid, DL-dihydrosphingosine 1, asparagine 3, alpha-tocopherol, ribonic acid, gamma-lactone, N-Acetyl-D-galactosamine 1, arachidic acid, cellobiose 2, 4-aminobutyric acid 1, elaidic acid, and conduritol b epoxide 2 were negatively associated with the levels of serum IL-1α, MIG, TNF-α, granzyme B, MCP-1, rantes, IL-1β, IL-9, fractalkine, IL-8, MIP-1β, IgE, and IL-6, but were positively associated with the levels of serum angiogenin, MCP-1α, and G-CSF.

### Influence of key microbial phylotypes on serum CRP levels and DS-related behaviors in germ-free mice

**Figure 7:**
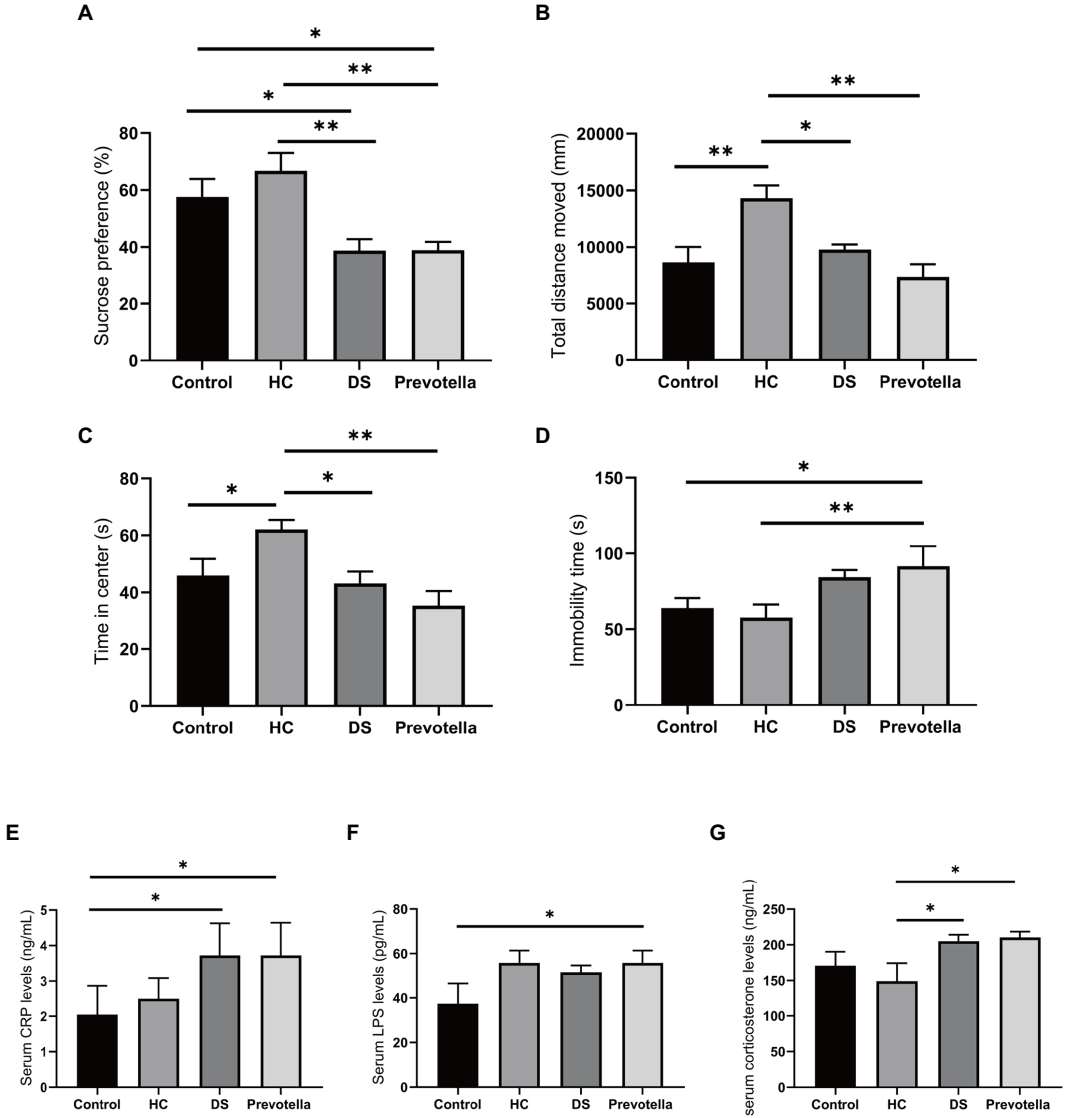
Behavioral effects of intestinal microbiota derived from non-DS volunteers or individuals with DS and *Prevotella copri* in germ-free mice. **(A–D)** Behavior of mice treated with intestinal microbiota derived from non-DS volunteers or individuals with DS and *Prevotella copri* in sucrose preference, open field, and forced swimming tests. Mice treated with intestinal microbiota derived from individuals with DS or *Prevotella copri* exhibited reduced sucrose preference **(A)**, reduced total distance traveled (open field test) **(B)**, reduced time in center (open field test) **(C)**, and increased immobility time (forced swimming test) **(D)**. **(E–G)** Effects of intestinal microbiota derived from non-DS volunteers or individuals with DS and *Prevotella copri* on serum CRP **(A)**, LPS **(B)**, and corticosterone **(C)** levels. Each group contained 6 mice. The data are expressed as the mean ± SEM from one-way ANOVA followed by a *post hoc* test and Student’s *t*-test. *p < 0.05, and **p < 0.01.

Previous studies have suggested that brain function and behavior can be affected by the gut flora. Therefore, we treated germ-free mice with the gut microbiota of non-DS volunteers or individuals with DS and a selected microbial species, *Prevotella copri*, to assess the effect on specific behaviors in mice. We selected *Prevotella copri* based on our consistent finding that the microbiome of individuals with DS is highly enriched in *Prevotella* bacteria compared to the microbiome of non-DS volunteers. Compared with the untreated control group, mice treated with fecal bacteria derived from individuals with DS exhibited reduced sucrose preference, reduced total distance moved and time in center of the open field test, and increased immobility time in the forced swimming test (all *p* < 0.01) (Figures 7A–D). In contrast, mice treated with feces derived from non-DS volunteers showed an increase in activity in the open field tests compared to controls (*p* < 0.01), an effect that needs to be further studied in the future. *Prevotella copri* treatment led to a significant reduction in sucrose preference, total distance moved, and time in center, and a significant increase in the immobility time, similar to the effect of the DS gut microbiota (all *p* < 0.01). The distance moved and time in center of mice in the *Prevotella* group were slightly lower than that of mice in the DS gut microbiota-treated group. These data suggest that the higher abundance of *Prevotella copri* in the microbiome of individuals with DS may play a role in modulating behaviors associated with DS.

Serum collected after the behavior tests was used to measure the levels of several microbial metabolites shown to be enriched in individuals with DS. LPS can attack multiple organs to promote inflammation and is used to establish animal models of cognitive impairment (Zhao et al., 2022). CRP is regarded as a marker of inflammation and corticosterone can maintain homeostasis through significant regulation of inflammation (Reeder and Kramer, 2005; Belda et al., 2020). Serum CRP, LPS, and corticosterone levels were significantly increased in mice treated with the intestinal microbiome derived from individuals with DS, suggesting that fecal bacteria play a role in DS (Figures 7E–G). Unexpectedly, we found an increase in serum LPS in the mice treated with the microbiome derived from non-DS volunteers, whereas CRP and corticosterone levels were not affected, a finding which needs to be addressed in the future. *Prevotella copri* treatment also significantly elevated serum CRP, LPS, and corticosterone levels in mice. These data suggest that changes in these metabolites may be correlated with specific behaviors in individuals with DS.

## Discussion

Down syndrome is one of the most common genetic causes of intellectual disability, with comorbid conditions that can reduce the quality of life and elevate the financial burden of care. Accumulating evidence suggests that intestinal microbiota play a vital role in various diseases and conditions and may serve as useful biomarkers of associated physiologic and behavioral changes (Choi et al., 2021). In this study, we found that serum pro-inflammatory cytokines, intestinal microbiota composition and diversity, and fecal metabolites were significantly different in individuals with DS compared to a group of volunteers without DS.

Dysbiosis of intestinal microbiota occurs frequently in individuals with DS and has been regarded as a contributor to comorbid conditions associated with DS (Ren et al., 2022). Previous studies have suggested that the reduction of microbiota diversity, as indicated by a reduction in the Shannon index and increase in the Simpson index at the OTU level, is associated with various neurodevelopmental and neurodegenerative disorders including autism, Alzheimer’s disease, and Parkinson’s disease (Harach et al., 2015; Hu et al., 2016; Mangiola et al., 2016). In the present study, we observed significantly different species diversity (per the Shannon index) in individuals with DS compared with non-DS volunteers, which may be associated with cognitive impairment in individuals with DS as suggested by others (Ren et al., 2022). Specifically, we found that the *Firmicutes/Bacteroidetes* ratio at the phylum level is significantly elevated in individuals with DS. This observation is consistent with clinical evidence suggesting that the alteration of intestinal microbiota in individuals with DS influences cognitive function by shifting the status of the microglia, such as through an increase in *Firmicutes* abundance (Jimenez et al., 2008). A high *Firmicutes/Bacteroidetes* ratio has also been reported in disorders of glycolipid metabolism, which may underlie developmental delays in individuals with DS (Grigor’eva, 2020). In addition, an increase in the *Firmicutes/Bacteroidetes* ratio has been linked to inflammation in patients with primary biliary cholangitis (Han et al., 2022). Therefore, the *Firmicutes/Bacteroidetes* ratio may be useful as a diagnostic biomarker for nutritional malabsorption in individuals with DS.

At the genus level, we found an abundance of *Prevotella* in the microbiome of the DS group compared to that of the non-DS group, where *Bacteroides* accounted for the largest proportion. These data are different from those in a previous report suggesting a higher abundance of *Parasporobacterium* and *Sutterella* in DS (Biagi et al., 2014). The discrepancy may be due to differences in demographic or lifestyle factors in study cohorts or differences in experimental conditions. We predict that even under the same living environment, the intestinal microbiota of those with DS and those without might be different. A larger sample size needs to be studied in the future. Nevertheless, it has been shown that eating patterns may regulate the gut microbiome by altering the use of nutrients (Wang and Kasper, 2014; Jiang et al., 2017). Both *Prevotella* and *Bacteroides* have been suggested as biomarkers for diet or disease (Gorvitovskaia et al., 2016). *Bacteroides* is well adapted to use of a large number of dietary polysaccharides and host-derived polysaccharides (such as mucus) (Martens et al., 2008). In the genome of *Bacteroides*, polysaccharide utilization sites have been widely expanded, and each seems to be dedicated to the utilization of specific categories of carbohydrates (Sonnenburg et al., 2010). The significant enrichment of *Bacteroides* in non-DS volunteers compared to individuals with DS in our study suggests a difference in carbohydrate intake between the two groups. *Prevotella copri* has previously been reported to induce insulin resistance and increase the risk of cardiovascular disease (Pedersen et al., 2016). *Prevotella copri* encodes superoxide reductase and adenosine phosphate phosphoryl sulfate reductase, both of which can enhance its resistance to ROS produced by inflammation, promote its proliferation in the inflammatory environment, and intensify inflammation (Scher et al., 2013). *Bacteroides* lacks these two enzymes (Scher et al., 2013), and its growth is inhibited and its abundance decreases in the inflammatory environment. The overexpression of SOD-1 gene in the 22.2–22.3 region of the long arm of chromosome 21 in individuals with DS (Donato, 2001; Netto et al., 2004) likely disturbs the steady-state biochemical balance of intracellular reactive oxygen species and increases the content of intracellular ROS, favoring enrichment of *Prevotella*.

We also found that *Roseburia, Anaerostipes*, and *Parasutterella* were reduced in individuals with DS compared to non-DS volunteers. *Roseburia* plays a vital role in the decomposition of polysaccharides to offer short-chain fatty acids to the host, enhance the body’s immune function, and regulate glycolipid metabolism (Du et al., 2020; Furuta et al., 2021). *Anaerostipes* maintains gut homeostasis through production of butyrate, the primary source of bacterial energy (Bui et al., 2014). *Parasutterella* serves as a core member of the intestinal microbiota, and its abundance is positively associated with the levels of short-chain fatty acids (SCFAs), which promote the secretion of IL-10 by regulating G-protein coupled receptor 43 (GPR43), a receptor for short-chain free fatty acids that is involved in the inflammatory response and regulation of lipid plasma levels (Sun et al., 2018; Mei et al., 2020). On the contrary, the relative abundance of *Escherichia/Shigella* and *Catenibacterium* was significantly higher in the DS group. *Escherichia/Shigella* are the mammalian bacillary dysentery pathogens, which result in diarrhea, fever, urinary tract infections, and pneumonia (Sun et al., 2019). *Catenibacterium* is Gram-positive bacteria, and its abundance is positively associated with serum biochemical parameters related to obesity and metabolic syndrome (Gallardo-Becerra et al., 2020). In addition, *Allisonella* genera are related to a pro-inflammatory phenotype (Aranaz et al., 2021). We found that increased serum pro-inflammatory cytokines was associated with a profile shift of gut microbiota in individuals with DS. Persistent and high-intensity inflammation in the intestines of individuals with DS may be related to their shift of microbiota composition. Therefore, enrichment of these microbial flora may be useful as diagnostic biomarkers or therapeutic targets for reducing gut inflammation in individuals with DS.

Since the fecal metabolome is closely related to the composition of intestinal microbiota, we performed GC-TOF-MS–based metabolomics analyses and found significant differences in fecal metabolites between individuals with DS and non-DS volunteers. Specifically, fecal behenic acid, α-tocopherol, DL- dihydrosphingosine, and aminobutyric acid levels were all significantly reduced in individuals with DS, while norvaline, glutaraldehyde, and glutaric acid were significantly increased compared to the non-DS group. Behenic acid is a saturated, very long-chain fatty acid that is beneficial for ameliorating postprandial inflammation by regulating serum IL-6, LPS, CRP, and insulin (da Silva et al., 2019). α-Tocopherol is a lipid-soluble antioxidant that is easily absorbed by the intestine, and plays a vital role in preventing oxidative damage to polyunsaturated fatty acids (Minehira-Castelli et al., 2006). Oral administration of DL-dihydrosphingosine inhibits the development of inflammation by protecting against TNF-α–induced cytotoxicity (Meyer and de Groot, 2003). Aminobutyric acid is an amino acid that ameliorates abnormal glycolipid metabolism in streptozotocin-induced diabetic mice (Abdelazez et al., 2022). Norvaline is a non-proteinogenic amino acid that destroys mitochondrial morphology and function by reducing cell viability at low concentrations (Samardzic and Rodgers, 2019). Glutaraldehyde is a five-carbon dialdehyde that is irritating to skin, eyes, nose, lungs, and increases the rates of cutaneous (Zissu et al., 1998). Glutaric acid, a water-soluble dicarboxylic acid, is one of the main metabolites of *Oscillibacter*. Glutaric acid stimulates the secretion of pro-inflammatory cytokines (Schmidt and Ferger, 2004; Athankar et al., 2017). Together, the observed differences in fecal metabolites in individuals with DS are linked not only to the metabolism dysfunction but also activation of the immune response and inflammation.

It has been previously reported the serum pro-inflammatory cytokines (including IL-2, IL-6, IL-8, IL-18, IL-1α, IL-1β, and TNF-α) are significantly increased and anti-inflammatory cytokines (including G-CSF) are significantly reduced in individuals with DS (Huggard et al., 2020; Martini et al., 2022). Consistent with these studies, we observed higher amounts of pro-inflammatory cytokines (including IL-1α, IL-1β, IL-6 and TNF-α) and lower anti-inflammatory cytokines (including G-CSF and angiogenin) in individuals with DS compared to non-DS volunteers. IL-1α is a canonical alarmin that induces neutrophil influx and activation, monocyte recruitment, prostaglandin synthesis, T- and B-cell activation and cytokine production by triggering the IL-1R, and results in the rapid recruitment of inflammatory cells (Batista et al., 2020). IL-1β is known as a key pro-inflammatory mediator that stimulates the NF-κB pathway (Karnam et al., 2020; Guo et al., 2021). In addition, increases in MCP-1 secretion are strongly associated with IL-1β and can activate multiple inflammatory pathways leading to the secretion of IL-6, TNF-α, and G-CSF (Huang et al., 2022). IL-6 is a well-established inflammation-promoting cytokine secreted by a variety of cell types (Mauer et al., 2015; Lin et al., 2021). Abnormal levels of TNF-α disturb the immune system by activating TNF receptors and up-regulating the downstream pathways and regulatory molecules, including NF-κB, MAPKs, caspases, and ROS/RNS (Blaser et al., 2016). In contrast, a high concentration of G-CSF is beneficial for promoting the survival, proliferation, differentiation, and function of granulocyte precursors (Cai et al., 2017). Furthermore, angiogenin has been reported to control bacterial overgrowth and maintain the integrity of the intestinal barrier (Chiang, 2017). We also found significant differences in glycolytic metabolic pathways between individuals with DS and non-DS volunteers. Parallel to the enrichment of *Prevotella* in individuals with DS, *Prevotella copri* intervention significantly increased the serum levels of CRP, LPS, and corticosterone in mice, which are all involved in inflammation. LPS can attack multiple organs, including liver, kidney, lung, and brain, to promote inflammation and is widely used to establish animal models of cognitive impairment, such as Parkinson’s disease (Zhao et al., 2022). CRP is regarded as a marker of inflammation that is strongly associated with the expression of adhesion molecules and chemokines in human endothelial cells. Corticosterone is the end product of the hypothalamic pituitary adrenal axis response to stress; it supports metabolic activities requiring energy to overcome environmental challenges and maintains homeostasis through significant regulation of inflammation (Reeder and Kramer, 2005; Belda et al., 2020). Therefore, there is a strong correlation between the observed DS-specific patterns of microbiota, metabolites, and inflammation.

Intestinal microbes and the brain interact with each other, and coordinate processes involved in health and disease (Ding et al., 2019). The gut–brain axis is a complex two-way system that uses various communication pathways, including endocrine, immune, neurotransmitter system, vagus nerve, and metabolites, such as SCFAs, branched chain amino acids, and peptidoglycans. In our studies on the effect of gut microbiota on behavior in mice, we found that the intestinal microbiota derived from individuals with DS and *Prevotella copri* could significantly change sugar water preference, autonomous activity, and depressive behavior. For rodents, the body’s receptors for sugar mainly exist in the oral cavity, which increase the body’s desire for sugar after being stimulated, and this desire is further enhanced in the intestinal tract (Sclafani and Ackroff, 2012). A low level of sugar water preference is often used as an indicator for depression (Lamers et al., 2019). In this study, the preference for sugar water was significantly lower in mice treated with intestinal flora from individuals with DS of *Prevotella copri* compared to untreated controls. In addition, we used open field test to evaluate the exploration ability and anxiety of experimental animals (Sheppard et al., 2022). We found that the movement distance and distance from the center of the open field were lower for mice transplanted with flora from individuals with DS and *Prevotella copri*, suggesting multiple systemic disorders related to basic motor skills, movement disorders, and abnormal gait and posture (Horvat et al., 2010), which are correlated with cognitive limitations, biomechanical defects, neurological defects, abnormal sensorimotor integration, and/or impaired somatosensory system found in individuals with DS (Carvalho and Vasconcelos, 2011). These data are consistent with the role of *Prevotella* in inflammatory diseases and its association with the development of cognitive impairment reported previously (Dillon et al., 2014; Wen et al., 2017). Therefore, we speculate that chronic inflammation and developmental delay in individuals with DS may be associated with the intestinal microbiota composition.

## Conclusion

The present study shows abnormal levels of serum cytokines, disordered intestinal microbial composition and diversity, and dysregulation of fecal metabolites in individuals with DS compared to non-DS volunteers. Since intestinal microbiota play a harmful role in the development of inflammation, intestinal microbiota and fecal metabolites-based biomarkers may provide potential targets for diagnosing and treating chronic inflammation in individuals with DS. This concept needs further clinical validation and mechanistic studies, in particular, a deeper exploration of the relationship between the intestinal microbiota and fecal metabolites and the levels of inflammation in individuals with DS.

## Data availability statement

The datasets presented in this study can be found in online repositories. The names of the repository/repositories and accession number(s) can be found at: NCBI – PRJNA870642.

## Ethics statement

The animal study was reviewed and approved by the Chinese Academy of Medical Sciences [Permit No. SYXK (Beijing)-2018-0019].

## Author contributions

SC, QC, YandingZ, and JH conceived and designed the experiments. SC, JL, ZL, SL, ZF, and YangfanZ performed the experiments and analyzed the data. SC and QC wrote the manuscript. All authors contributed to the article and approved the submitted version.

## Funding

This work was supported by the Natural Science Foundation of Fujian Province, China (Grant Nos. 2017J01621 and 2021J01202).

## Conflict of interest

The authors declare that the research was conducted in the absence of any commercial or financial relationships that could be construed as a potential conflict of interest.

## Publisher’s note

All claims expressed in this article are solely those of the authors and do not necessarily represent those of their affiliated organizations, or those of the publisher, the editors and the reviewers. Any product that may be evaluated in this article, or claim that may be made by its manufacturer, is not guaranteed or endorsed by the publisher.

## Footnotes

^1^ http://www.metaboanalyst.ca/

^2^ http://www.kegg.jp/kegg/pathway.html
