# Supplementary material for: Alterations in intestinal microbiota and metabolites in individuals with Down syndrome and their correlation with inflammation and behavior disorders in mice
Source: Front Microbiol. 2023 Feb 23;14:1016872. doi: 10.3389/fmicb.2023.1016872 (PMC9998045; doi:10.3389/fmicb.2023.1016872)
Supplement: Supplementary file 1 [file Data_Sheet_1.docx]

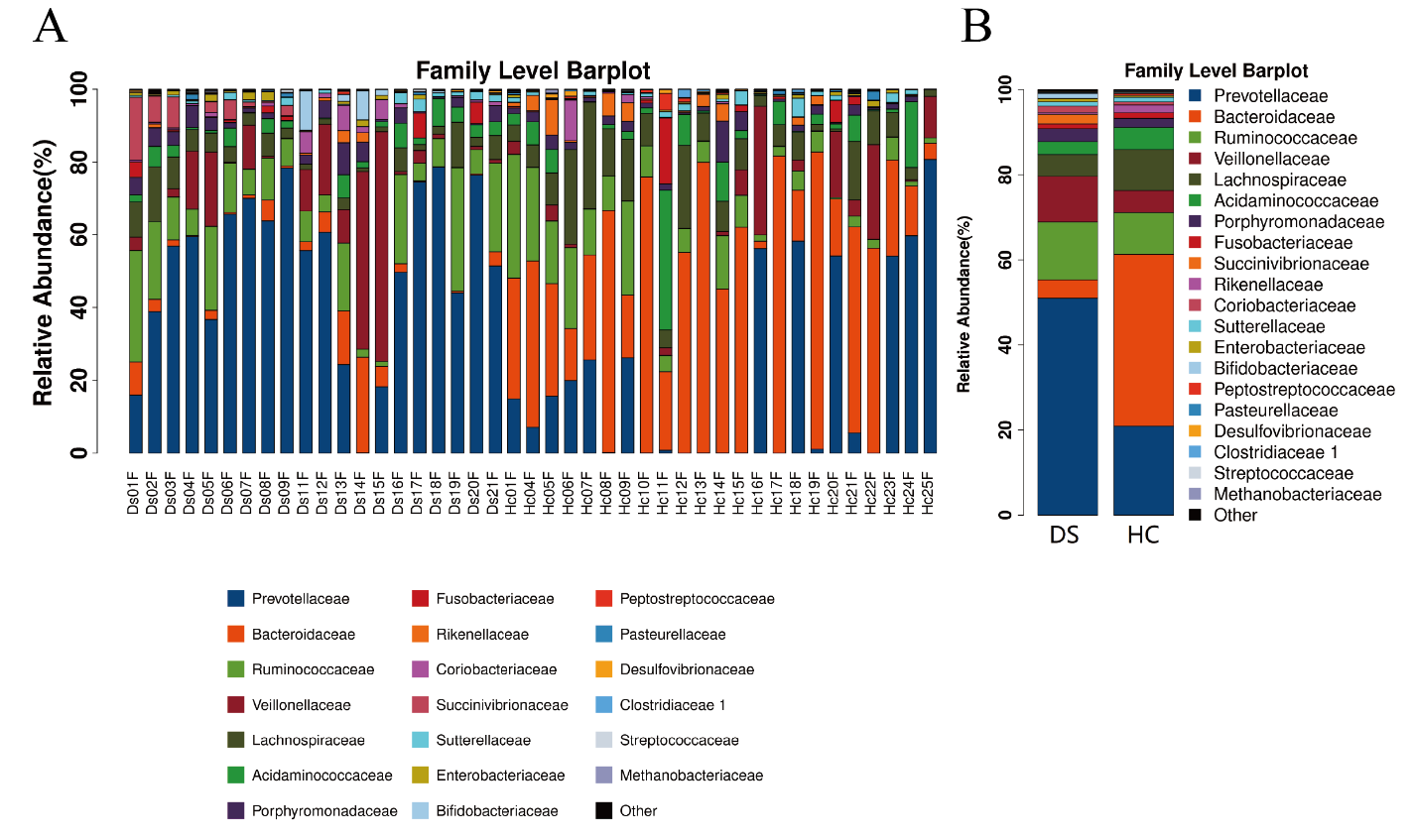


**Supplemental Figure 1.** Differences in microbial composition between individuals with DS (DS) and non-DS volunteers (HC) at the family level. *Prevotellaceae* was enriched in individuals with DS, followed by *Ruminococcaceae, Veillonellaceae, Lachnospiraceae*, and *Bacteroidaceae*. *Bacteroidaceae* was enriched in the non-DS group, followed by *Prevotellaceae, Lachnospiraceae, Ruminococcaceae,* and *Acidaminococcaceae*.

**
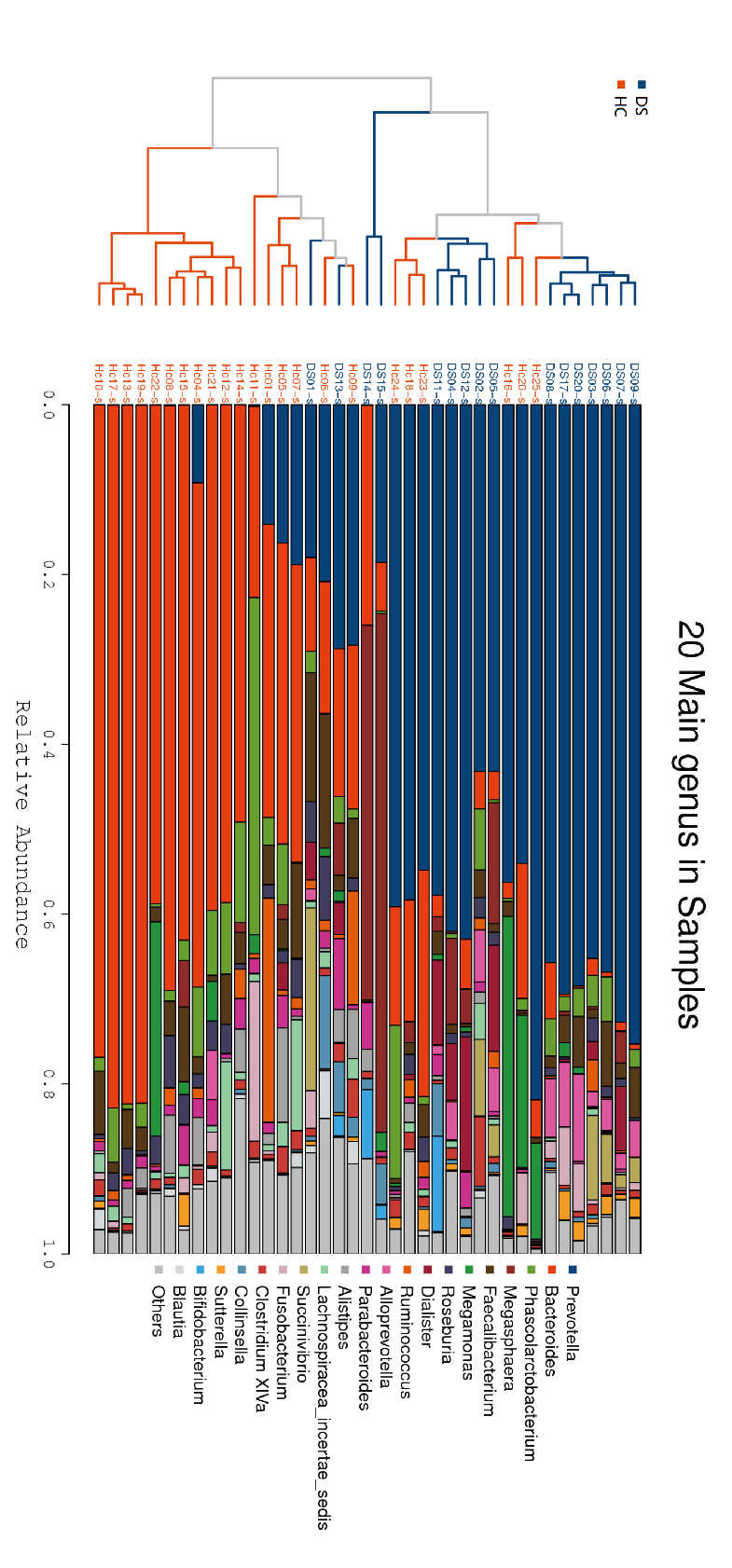
**

**Supplemental Figure 2.** Hierarchical clustering of gut flora of individuals with DS (DS) and non-DS volunteers (HC). The top 20 genera were compared. At the genus level, the fecal flora of individuals with DS and non-DS volunteers (HC) were clearly divided into two distinct groups (clusters).

**
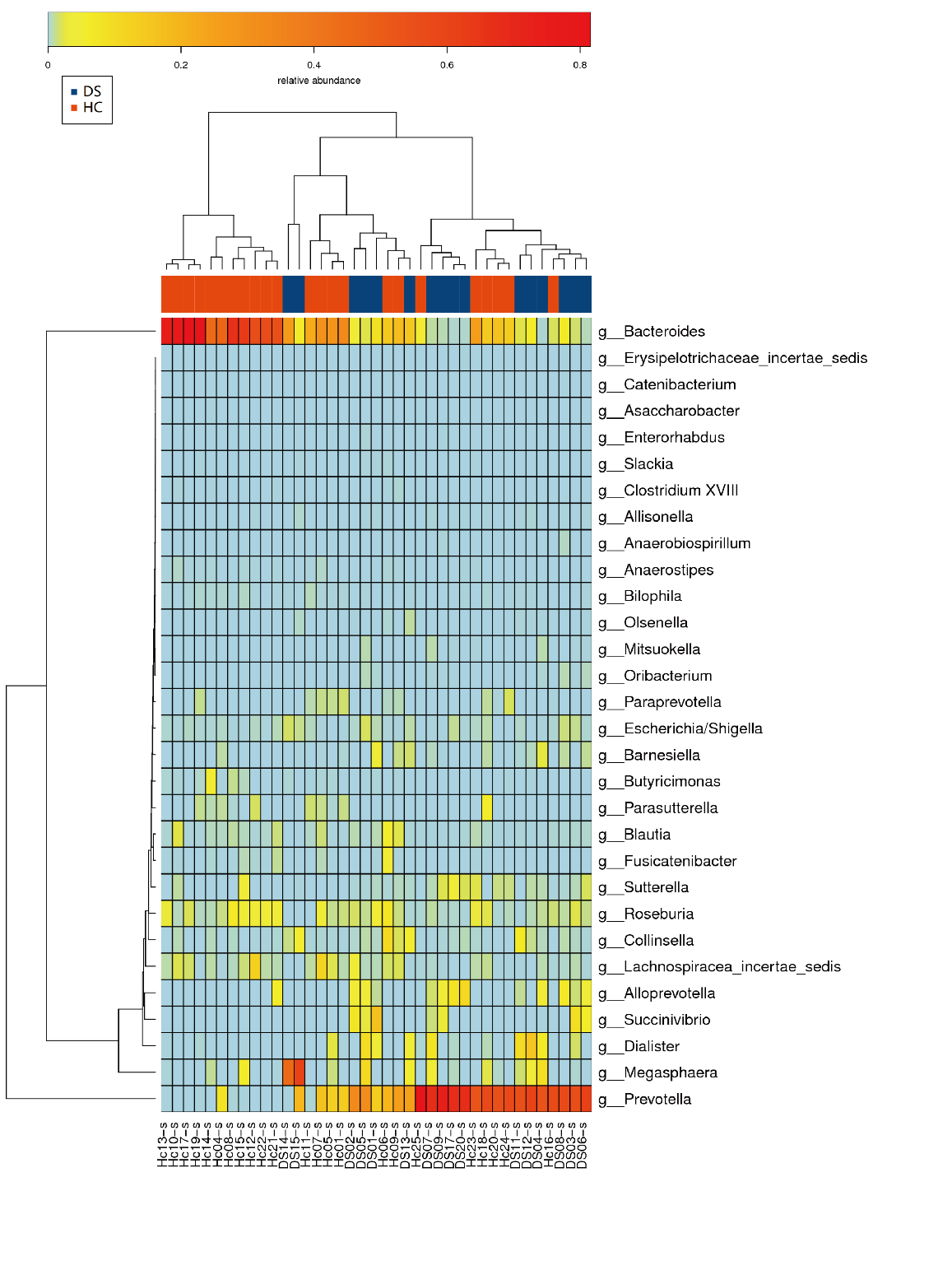
**

**Supplemental Figure 3.** Heatmap of the gut flora cluster analysis. The size of values in the data matrix are indicated by color gradient and clustered based on abundance in the sample. Significantly different flora between the two groups include *Clostridium XLVA, Parabacteroids, Roseburia, Alistipes, Collinsella, Bifidobacterium, Lachnospiracea incertae sedis, Alloprevotella, Fusobacterium, Dialist, Faecalibacterium, Succenivibrio, Ruminococcus, Phascolarctobacterium, Megamonas, Megasphaera, Bacteroides,* and *Prevotella*. *Prevotella* emerged as the dominant bacterium in individuals with DS (DS), whereas *Bacteroides* was dominant in non-DS volunteers (HC).

**
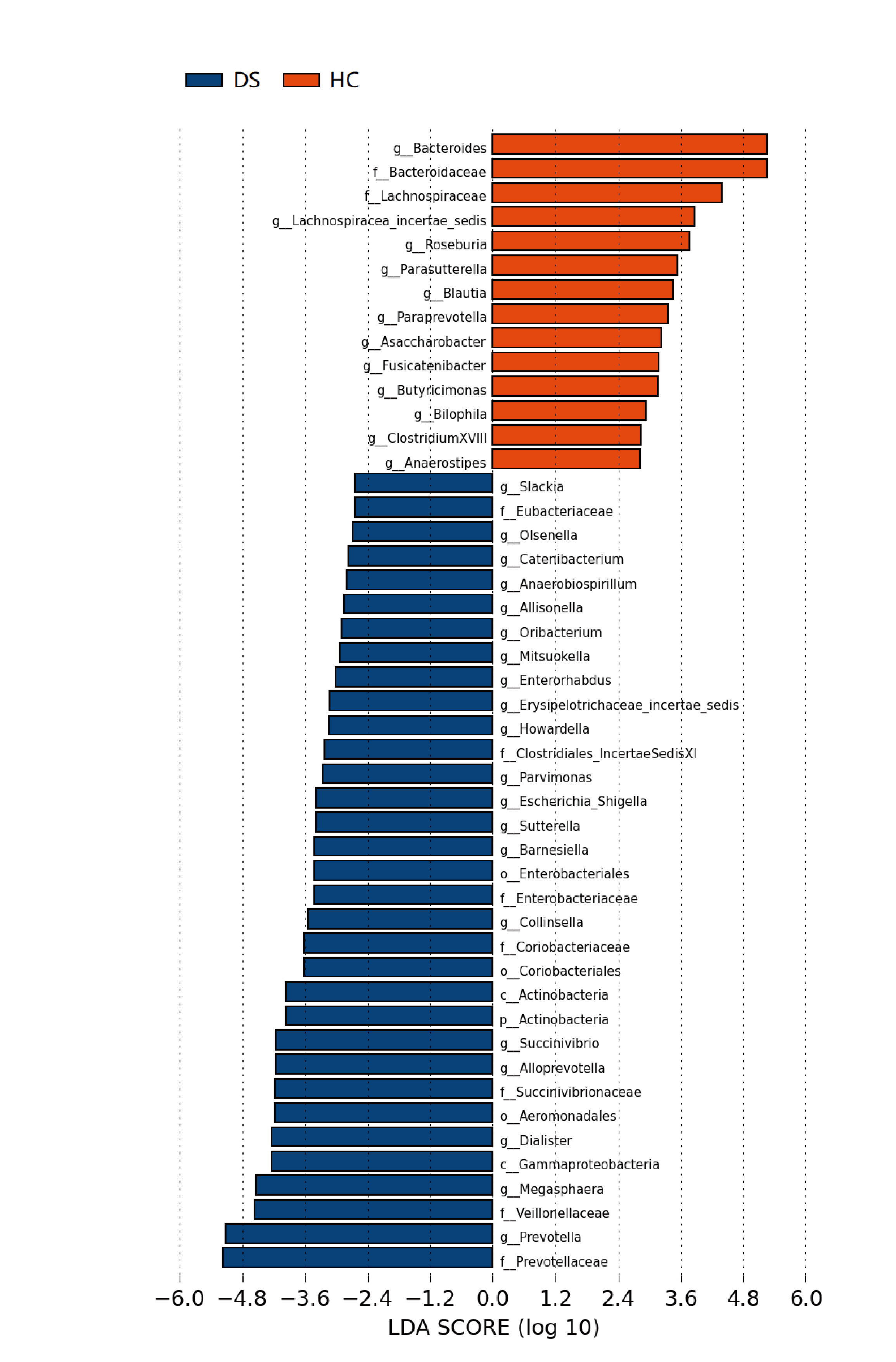
**

**Supplemental Figure 4.** LDA effect size (LEfSe) analysis was used to compare the DS and non-DS groups and identify subgroups within each group. Difference in abundance between these two groups was found, with the LDA score indicating the effect size and rank of differentially abundant taxon. The dominant bacteria in the non-DS group included *Bacteroides, Bacteroidaceae, Lachnospiracea, Lachnospiracea incertae sedis, Roseburia, Parasutterella, Blautia, Paraprevotella, Asaccharacter, Fusicatenibacter, Butyrimimonas, Bilophila, Clostridium XVIII,* and *Anaerostipe*. The following bacteria were significantly higher in individuals with DS: *Slackia, Eubacteriaceae, Olsenella, Catenibacterium, Anaerobiospirillum, Allisonella, Oribacterium, Mitsuokella, Enterorhabdus, Erysipelotrichaceae incertae sedis, Howardella, Clostridiales incertae sedis XI, Parvimonas, Escherichia/Shigella, Sutterella, Barnesiella, Enterobacteriales, Enterobacteriaceae, Collinsella, Coriobacteriaceae, Coriobacteriales, Actinobacteria, Succinivibrio, Alloprevotella, Succinivibrioaceae, Aeromonadales, Dialister, Gammaproteobacteria, Megasphaera, Veillonellaceae, Prevotella,* and *Prevotellaceae*.

**
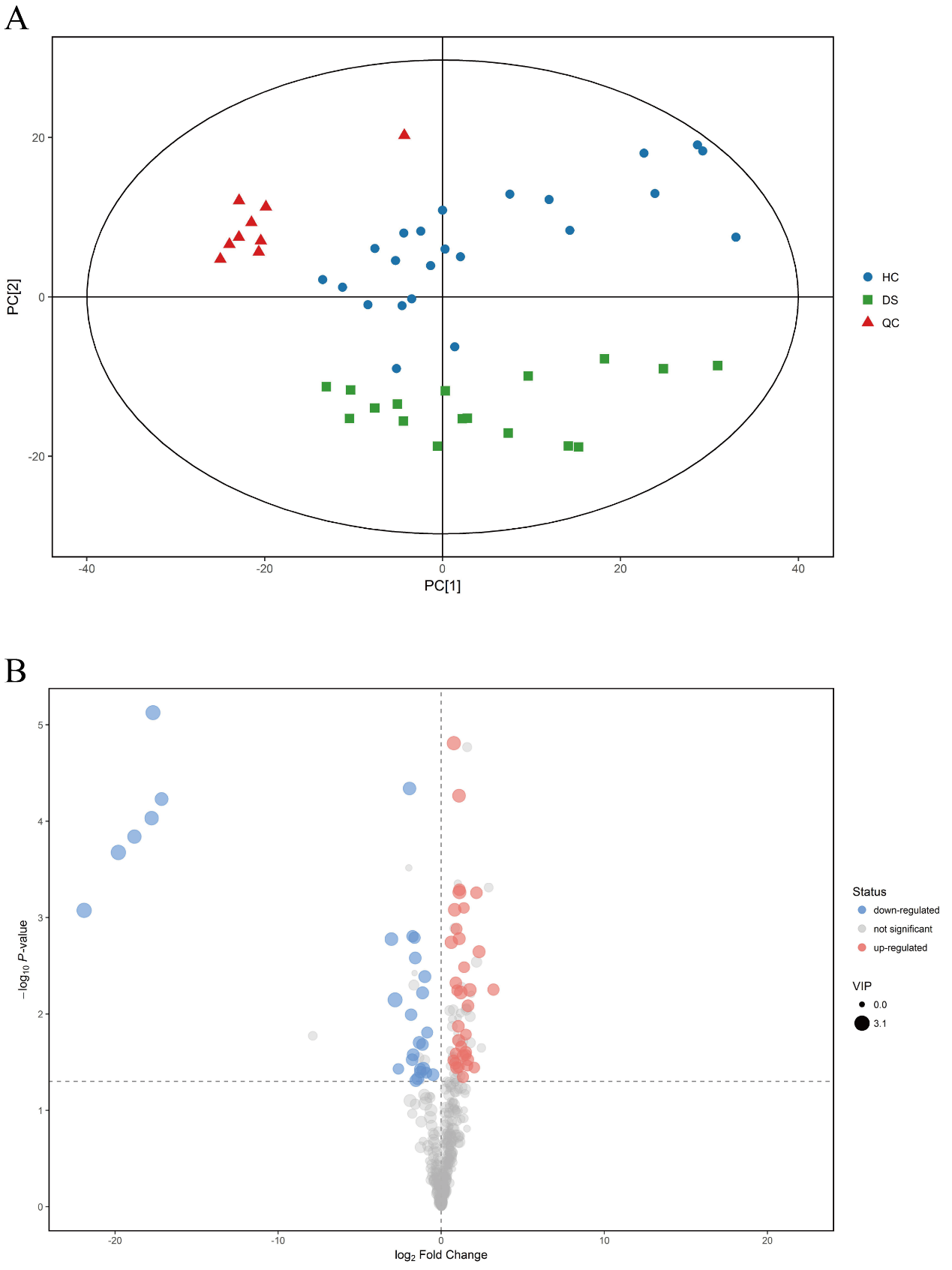
**

**Supplemental Figure 5.** PCA score scatter plot **(A)** and volcanic map **(B)** of all samples from individuals with DS and non-DS volunteers (HC), as well as QC samples, following GC-TOF-MS. **(A)** The abscissa PC[1] and ordinate PC[2] represent the scores of the first and second principal components, respectively, and the scatter color and shape represent the experimental grouping of samples. The distinction between groups is highly significant in the top ranked principal components, and the samples are all within the 95% confidence interval (Hotelling’s T-squared ellipse). **(B)** Volcano map was used to show the differences in all total metabolites. Each point represents a metabolite, the abscissa represents the multiple change of each substance in the group compared (take the logarithm based on 2), the ordinate represents the *p*-value of Student’s *t*-test (take the logarithm based on 10), and the scatter size represents the VIP value of OPLS-DA model. The larger the scatter, the greater the VIP value. A VIP value more than 1.0 and *P* value less than 0.05 were considered statistically significant. The fecal metabolites that are significantly higher in individuals with DS compared to non-DS volunteers are shown in red, metabolites that are significantly lower are shown in blue, and metabolites with no significant difference between the two groups are shown in gray.
