# Supplementary material for: Alterations in intestinal microbiota and metabolites in individuals with Down syndrome and their correlation with inflammation and behavior disorders in mice
Source: Front Microbiol. 2023 Feb 23;14:1016872. doi: 10.3389/fmicb.2023.1016872 (PMC9998045; doi:10.3389/fmicb.2023.1016872)
Supplement: Supplementary file 2 [file Data_Sheet_2.docx]

**Supplemental materials**

**Supplemental Table 1.** Characteristics of Individuals with Down syndrome (DS) and non-DS Volunteers (HC)

| Participant No. | Age | Sex |
| --- | --- | --- |
| DS01 | 14 | Male |
| DS02 | 45 | Male |
| DS03 | 29 | Male |
| DS04 | 21 | Female |
| DS05 | 27 | Female |
| DS06 | 39 | Female |
| DS07 | 28 | Female |
| DS08 | 14 | Female |
| DS09 | 14 | Female |
| DS11 | 4 | Female |
| DS12 | 5 | Female |
| DS13 | 11 | Male |
| DS14 | 16 | Female |
| DS15 | 19 | Male |
| DS16 | 37 | Female |
| DS17 | 31 | Male |
| DS18 | 26 | Female |
| HC01 | 38 | Male |
| HC02 | 36 | Female |
| HC03 | 21 | Female |
| HC04 | 7 | Female |
| HC05 | 10 | Female |
| HC06 | 25 | Male |
| HC07 | 29 | Female |
| HC08 | 43 | Male |
| HC09 | 12 | Female |
| HC10 | 27 | Female |
| HC11 | 22 | Female |
| HC12 | 51 | Female |
| HC13 | 23 | Male |
| HC14 | 27 | Female |
| HC15 | 19 | Female |
| HC16 | 17 | Female |
| HC17 | 22 | Female |
| HC18 | 16 | Female |
| HC19 | 18 | Male |
| HC20 | 23 | Female |
| HC21 | 14 | Male |
| HC22 | 29 | Female |

**Supplemental Table 2.** Alpha Diversity Analysis of Gut Microbiota in Individuals with DS and non-DS Volunteers (HC)

| Group | Chao | Simpson | Shannon | Coverage |
| --- | --- | --- | --- | --- |
| DS | 188.41 | 0.8447 | 4.1128 | 0.9986 |
| HC | 143.42 | 0.8067 | 3.7086 | 0.9989 |

**Supplemental Table 3.** Differences in Fecal Metabolites in the non-DS and DS Groups

| ID | Compound | R.T. | Mass | Mean DS | Mean HC | VIP | P-value | FC |
| --- | --- | --- | --- | --- | --- | --- | --- | --- |
| ***1*** | ***Tyramine*** | ***18.27*** | ***174*** | ***0.49*** | ***0.05*** | ***1.37*** | ***0.01*** | ***9.18*** |
| 2 | Methionine sulfoxide 2 | 16.89 | 128 | 0.02 | 0.00 | 1.73 | 0.00 | 4.98 |
| ***3*** | ***3-Hydroxybutyric acid*** | ***8.98*** | ***147*** | ***1.45*** | ***0.43*** | ***2.05*** | ***0.01*** | ***3.36*** |
| 4 | Oxamic acid | 10.28 | 147 | 0.02 | 0.01 | 1.62 | 0.03 | 3.08 |
| 5 | Oxalacetic acid | 13.16 | 259 | 0.01 | 0.00 | 1.23 | 0.03 | 3.04 |
| 6 | Farnesal 1 | 16.50 | 167 | 0.07 | 0.03 | 1.62 | 0.02 | 2.82 |
| 7 | Cycloserine | 15.54 | 234 | 0.01 | 0.00 | 1.10 | 0.00 | 2.62 |
| 8 | Glutaraldehyde 2 | 8.95 | 86 | 0.06 | 0.03 | 2.14 | 0.01 | 2.29 |
| 9 | Phenylacetic acid | 11.00 | 164 | 0.54 | 0.25 | 2.18 | 0.00 | 2.17 |
| 10 | Phenylalanine 1 | 15.14 | 218 | 0.87 | 0.40 | 1.57 | 0.00 | 2.17 |
| 11 | Isoleucine | 10.88 | 158 | 1.73 | 0.81 | 2.10 | 0.00 | 2.14 |
| 12 | Norvaline | 10.11 | 144 | 0.05 | 0.02 | 1.70 | 0.02 | 2.10 |
| 13 | Indole-3-acetamide 2 | 18.84 | 318 | 0.00 | 0.00 | 1.19 | 0.04 | 2.10 |
| 14 | Benzyl alcohol | 8.92 | 165 | 0.63 | 0.31 | 1.61 | 0.01 | 2.06 |
| 15 | 4-Methyl-5-thiazolethanol | 11.96 | 103 | 0.01 | 0.01 | 1.18 | 0.01 | 1.97 |
| 16 | Pipecolinic acid | 11.86 | 156 | 0.70 | 0.38 | 1.57 | 0.00 | 1.86 |
| 17 | Beta-Alanine 2 | 12.68 | 248 | 0.04 | 0.02 | 1.16 | 0.03 | 1.85 |
| 18 | Valine | 9.78 | 144 | 2.19 | 1.24 | 2.02 | 0.00 | 1.76 |
| 19 | Alanine 1 | 8.16 | 116 | 5.29 | 3.09 | 2.33 | 0.00 | 1.71 |
| ***20*** | ***Glutaric Acid*** | ***12.39*** | ***147*** | ***0.38*** | ***0.23*** | ***1.19*** | ***0.03*** | ***1.68*** |
| 21 | Serine 1 | 11.77 | 204 | 0.69 | 0.45 | 1.92 | 0.00 | 1.54 |
| 22 | N-Acetyl-D-galactosamine 1 | 19.82 | 202 | 0.08 | 0.15 | 1.66 | 0.00 | 0.50 |
| 23 | 4-Aminobutyric acid 1 | 13.98 | 174 | 0.05 | 0.10 | 1.98 | 0.04 | 0.47 |
| 24 | Cellobiose 2 | 24.98 | 361 | 0.03 | 0.08 | 1.52 | 0.04 | 0.41 |
| 25 | Conduritol b Epoxide 2 | 18.64 | 217 | 0.01 | 0.03 | 1.73 | 0.05 | 0.37 |
| 26 | Alpha-Tocopherol | 28.25 | 237 | 0.01 | 0.03 | 1.70 | 0.05 | 0.34 |
| 27 | Ribonic acid， gamma-lactone | 15.80 | 117 | 0.01 | 0.02 | 1.61 | 0.00 | 0.33 |
| 27 | 3-Cyanoalanine | 12.04 | 227 | 0.00 | 0.00 | 1.42 | 0.00 | 0.32 |
| 29 | Asparagine 3 | 15.03 | 257 | 0.00 | 0.01 | 1.52 | 0.03 | 0.29 |
| 30 | Elaidic acid | 21.30 | 337 | 0.00 | 0.02 | 1.51 | 0.01 | 0.28 |
| 31 | Alpha-Aminoadipic acid | 16.13 | 154 | 0.02 | 0.06 | 2.02 | 0.00 | 0.26 |
| ***32*** | ***Arachidic acid*** | ***23.05*** | ***117*** | ***0.04*** | ***0.31*** | ***2.86*** | ***0.01*** | ***0.14*** |
| ***33*** | ***1-Monopalmitin*** | ***24.15*** | ***371*** | ***0.00*** | ***0.01*** | ***2.80*** | ***0.00*** | ***0.00*** |
| ***34*** | ***DL-dihydrosphingosine 1*** | ***23.48*** | ***204*** | ***0.00*** | ***0.05*** | ***3.06*** | ***0.00*** | ***0.00*** |
| ***35*** | ***Behenic acid*** | ***24.58*** | ***117*** | ***0.00*** | ***0.22*** | ***2.99*** | ***0.00*** | ***0.00*** |

Note: Red represents metabolites that are higher and blue represents metabolites that are lower in fecal samples from individuals with DS compared to non-DS volunteers.

Bold italics indicate a close correlation between the fecal metabolite and characteristic bacteria identified in the intestinal microbiome of individuals with DS.
